# Reliability and Validity of the European Portuguese Version of the Social Touch Questionnaire

**DOI:** 10.1007/s10919-016-0239-7

**Published:** 2016-07-15

**Authors:** Ana Isabel Vieira, Ana Vanessa Ramos, Luís Manuel Cavalheiro, Patrícia Almeida, Dália Nogueira, Elisabeth Reis, Maria Vânia Nunes, Alexandre Castro-Caldas

**Affiliations:** 1 Alcoitão School of Health Sciences, Rua Conde Barão, Alcoitão, 2649-506 Alcabideche, Portugal; 2 CEISUC - Centre of Study and Research in Health, University of Coimbra, Avenida Dias da Silva, 165, 3004-512 Coimbra, Portugal; 3 Business Research Unit, ISCTE-University Institute of Lisbon, Avª das Forças Armadas, 1649-026 Lisbon, Portugal; 4 Institute of Health Sciences, Catholic University of Portugal, Palma de Cima, 1649-023 Lisbon, Portugal

**Keywords:** Social touch, Social anxiety, Social Touch Questionnaire, Cultural adaptation, Reliability, Validity, Exploratory and confirmatory factor analyses

## Abstract

Social touch is essential for physical and emotional well-being. However, different meanings can be attributed to physical contact during social interactions and may generate bonding or avoidant behaviors. This personal and unique experience is not usually taken into account in health and social care services. The aim of this study is to produce a valid and reliable European Portuguese version of the Social Touch Questionnaire (STQ, Wilhelm et al. in Biol Psychol 58:181–202, 2001. doi:10.1016/S0301-0511(01)00113-2). The STQ is a self-report questionnaire for adolescents and adults measuring behaviors and attitudes towards social touch. The original version was translated into European Portuguese using a forward-back translation process and its feasibility was examined. To evaluate the psychometric properties, a total of 242 Portuguese university students participated in the study (21.3 ± 3.8 years). The STQ was considered feasible, showed adequate internal consistency (Cronbach’s α = .734), and the test–retest correlation with the STQ items demonstrated a high concordance between the tests over a two-week interval (ICC = .990; *n* = 50). Validity tests were performed, comparing the total score of the STQ with that of the anxiety and avoidance subscales of the Social Interaction and Performance Anxiety and Avoidance Scale (SIPAAS). A very significant conceptual convergence was confirmed between the STQ and with the SIPAAS-Anxiety (*r* = .64; *p* < .0001) and with the SIPAAS-Avoidance (*r* = .59; *p* < .0001). The exploratory factor analysis, with Promax rotation, revealed 3 factors: dislike of physical touch, liking of familiar physical touch and liking of public physical touch (Cronbach’s alphas ranged from .68 to .75). Psychometric properties confirmed the adaptation of the STQ to the Portuguese culture. It is a reliable and valid self-report questionnaire and it appears to be a useful tool to assess behaviors and attitudes towards social touch.

## Introduction

Touch is our first form of communication and probably the most important and universal form of human attachment bond. From the day we are born, we touch and are touched by others and the quality of this tactile interaction is determinant in neurodevelopment, and in the capability to transmit, control, and understand emotions. Moreover, it is crucial to learn how to cope with social interactions (Dunbar 2010). Social touch is a distinct domain of touch and is a fundamental human need, essential for our physical and emotional wellbeing (Olausson et al. 2010). It encompasses all the situations in which people touch each other (Haans et al. 2014; Jones and Brown 1996).

The “Social Touch Hypothesis” is based on pleaseant touch, since it mediates the communication and interpretation of affective contact during the interactions with others. C tactile (CT) afferents, together with Aβ afferents, support this theory and represent the neurobiological substrate of affective touch (McGlone et al. 2014), fostering empathic responses (Morrison et al. 2011) and therefore interpersonal touch, affiliative behavior, and social interaction (McGlone et al. 2012; Olausson et al. 2010).

Social touch-based contact can be categorized into (1) simple, if the touch has a short duration, is intentional and is applied on a restricted part of the body; (2) protracted, if touch involves longer and mutual contact (embrace or holding hands); (3) dynamic, if touch comprises continuous and repetitive movements over the skin (caressing) (Morrison et al. 2010). The differences in interpersonal touch can be influenced by intrinsic and extrinsic factors: (1) the use of touch in some cultures is perceived as warm and friendly while in others it is seen as intrusive and inappropriate (Wilson and Rockstraw 2012); (2) the specific body part where touch occurs and the specific characteristics of the person that touches (gender, age, and relationship with the touched person) (Gallace and Spence 2010); (3) emotional and psychological aspects of the recipient.^1^ Like all nonverbal behaviors, touch may have many interpretations or meanings and the above mentioned social touch categories are not always well received and the experience of being touched is not always pleasant.

Touch is a nonverbal variable in health care that can cause problems in therapeutic settings (Wilson and Rockstraw 2012) and touch avoidance is an indicator of a person’s attitude towards touch (Andersen 1999). Therefore, before any therapeutic intervention involving hands-on strategies,^2^ it is essential to assess the patient’s perception of touch. Moreover, the individual reactions of both the patient and the health professional must be continuously monitored. This entails discussing interventions to ensure a clear understanding of the therapeutic intent and the meaning of touch (Fosshage 2000).

To assess a patient perception of touch, we consider the Social Touch Questionnaire (STQ) by Wilhelm et al. (2001) to be the most appropriate instrument to measure social touch, as it evaluates a very comprehensive range of behaviors and attitudes toward touch and can be applied in various contexts and by different professionals in health, social, and educational areas. It is also the most likely to be adapted to the Portuguese culture because we have not found any instruments regarding touch perception specifically adapted to the Portuguese culture. Questionnaires designed to assess health and health outcomes from the patient’ point of view are of great importance (Feeney 2002), not only because they give health professionals insights to problems that are not consciously or verbally referenced by the patient but also because these problems may have a negative influence on the success of the intervention and can therefore influence the prognosis.

The translation and cultural adaptation of instruments facilitates research by academics and health professionals, making them more culturally appropriate and comparable across different populations. The adaptation and validation process aims to produce an instrument with the same comparable psychometric qualities as the original. This process is crucial because there may be some inconsistency between the culture and language of the original measurement instrument and the context in which it will be applied (Scientific Advisory Committee of the Medical Outcome Trust 2002; Terwee et al. 2007).

Other instruments to assess behaviors and attitudes towards touch described in literature did not fulfill our purposes of evaluating specifically social touch. For instance, (1) Touch Avoidance Questionnaire (TAQ) places particular emphasis on situations involving partners, parents, siblings, and friends as opposed to social touch (Ozolins and Sandberg 2009); (2) Andersen and Leibowitz Inventory Touch Avoidance Measurement (TAM) is designed to assess individual differences in the perception of touch behavior by a friend of the same or opposite sex (Andersen and Leibowitz 1978).

The aim of the current research was thus to produce a valid and reliable European Portuguese version of the STQ. This study followed the basic ethical principles set by the Declaration of Helsinki and we received prior approval from an institutional review board and all subjects gave their written informed consent. All subjects involved in the study signed a written informed consent for the usage of the data provided.

## Methods

This study was conducted in two phases: (phase 1) a cultural and linguistic adaptation of the STQ to Portuguese; (phase 2) a reliability and validity test of the version obtained in the first phase. Permission to carry out the translation and validation of the instrument was granted by from the authors of the original STQ (Wilhelm et al. 2001).

### Description of the Original Social Touch Questionnaire (STQ)

The STQ (Wilhelm et al. 2001) was designed to assess the behavior and attitudes towards social touch in a study of college students with social anxiety. The questionnaire consists of 20 items covering a wide range of issues concerning affections and attitudes towards social touch, such as touching versus being touched, touching someone you known versus touching a stranger, touching someone in a public place versus in a private place, touching without sexual connotation versus touching with sexual connotation.

Each subject is asked to state how far the statements are true using a Likert scale from 0 (*not at all*) to 4 (*extremely*). To obtain the total score, ten items with negative polarity need to be encoded in reverse (item 1, 4, 6, 9, 11, 12, 14, 15, 18 and 20), since they have negative polarity. The total score is thus obtained by summing the scores for each of the items; the spectral quantification of the total score goes from 0 (lowest avoidance of social touch) to 80 (most avoidance of social touch). The internal consistency [Cronbach’s Alpha (α)] of the overall questionnaire in the sample of the original study was .89, with an average item inter-correlation of .29.

### Phase 1—Cultural and Linguistic Adaptation

The process of forward and back translations began with the translation of the original version of STQ into Portuguese. This translation was performed independently by two bilingual Portuguese translators. A consensus version was then obtained by a panel of experts in order to examine the equivalence of meaning of the translated items and the quality of translation, namely with respect to clarity, colloquial language and literal translation. The back translation was performed by two translators whose native language is English, and a panel of experts then crosschecked these versions with the original questionnaire. Back translation was sent also to the authors of the original questionnaire and their opinions were taken into consideration. The semantic equivalence was then analyzed from the clinical point a view by two physiotherapists specialized in human behavior and neurology and with proven scientific work in the area of “Touch”. This led to the pre-final version of the questionnaire.

The content validity was examined to assess the clarity, understanding, cultural relevance, and the setting of the words used when applying the STQ by administering a comprehension test to a convenience sample of 20 adult individuals. The sample consisted of 10 finalists of a physiotherapy degree and 10 institutionalized individuals diagnosed with schizophrenia. This clinical condition was selected because its symptoms lead to changes in social functioning, (Sitzer et al. 2008) and the avoidance of contact with others. Students from the physiotherapy degree course were chosen because they are exposed to numerous situations where they have to touch and be touched and so they may exhibit fewer touch avoidance behaviors and attitudes.

Table 1 presents the characterization of the sample. The majority of participants were female (90 %) with a mean age of 39 ± 18.4 years (min = 21, max = 64) and a mean education of 14 ± 2.9 years (min = 9; max = 16). The average time taken to complete the questionnaire was 9.1 ± 6.9 min (min = 2, max = 23). Subjects with schizophrenia took much longer (15.3 ± 3.7; min = 11, max = 23) than the students (3 ± .8; min = 2, max = 4); this difference may be explained by the typical symptomatology of schizophrenia, namely disorganized thinking, cognitive deficits, deficit of attention, deficits of declarative and working memory, memory, language function, and slower planning of activities (American Psychiatric Association 2013).

**Table 1:** Sample characteristics (*N *= 20) and completion time of STQ

Individuals with schizophrenia (*n*; %)	10 (50 %)
Students (*n*; %)	10 (50 %)
Women (*n*; %)	18 (90 %)
Average age of sample (years)	39 ± 18.4 (21–64)^a^
Average patient age with schizophrenia (years)	56.8 ± 4.0 (50–64)^a^
Average age of students (years)	21,3 ± .4 (21–22)^a^
Education of the sample (years)	14 ± 2.9 (9–16)^a^
Education of individuals with schizophrenia (years)	12 ± 3.0 (9–16)^a^
Education of students (years)	16 ± .0 (16–16)^a^
STQ completion time (min)	9.1 ± 6.9 (2–23)^a^
Completion time by individuals with schizophrenia (min)	15.3 ± 3.7 (11–23)^a^
Completion time by students (min)	3 ± .8 (2–4)^a^

^a^Mean ± standard deviation (minimum–maximum)

All the participants (*n* = 20) were of the opinion that the STQ was a relevant questionnaire, explicit, noticeable, understandable, quick and easy to answer, and that the instructions were clear. The proposed solutions were reviewed by the panel of experts and analyzed for their responsiveness and adequacy. The European Portuguese version of the STQ resulted from consensus achieved amongst the panel of experts. The items of the Portuguese version following the cultural and linguistic adaptation are presented in Table 2.

**Table 2:** Items from the European Portuguese version of the STQ following the cultural and linguistic adaptation

Item	Original version	Portuguese version
1	I generally like it when people express their affection towards me in a physical way^a^	Normalmente gosto que as pessoas manifestem o seu afeto por mim de uma forma física^a^
2	I feel uncomfortable when someone I don’t know very well hugs me	Sinto-me pouco à vontade quando alguém que não conheço muito bem me dá um abraço
3	I get nervous when an acquaintance keeps holding my hand after a handshake	Fico nervoso(a) quando uma pessoa não larga a minha mão depois de um aperto de mão
4	I generally seek physical contact with others^a^	Normalmente procuro contato físico com os outros^a^
5	I feel embarrassed if I have to touch someone in order to get their attention	Sinto-me constrangido/a se tenho de tocar em alguém para chamar a sua atenção
6	I consider myself to be a ‘touchy-feely’ person^a^	Considero-me uma pessoa que gosta de expressar afeto através do toque^a^
7	It annoys me when someone touches me unexpectedly	Aborrece-me que alguém me toque inesperadamente
8	I’d feel uncomfortable if a professor touched me on the shoulder in public	Sentir-me-ia pouco à vontade se um professor me tocasse no ombro em público
9	I’d be happy to give a neck/shoulder massage to a friend if they are feeling stressed^a^	Teria todo o gosto em fazer uma massagem no pescoço ou nos ombros a uma pessoa amiga que estivesse tensa^a^
10	I feel uncomfortable if I make physical contact with a stranger on the bus or subway	Sinto-me pouco à vontade se tiver contato físico com um estranho no autocarro ou no metropolitano
11	I like being caressed in intimate situations^a^	Gosto de receber carícias em situações íntimas^a^
12	As a child, I was often cuddled by family members (e.g. parents, siblings)^a^	Quando era criança, os meus familiares (por exemplo, pais, irmãos) faziam-me festas muitas vezes^a^
13	I would rather avoid shaking hands with strangers	Preferiria evitar dar apertos de mão a estranhos
14	I greet my close friends with a kiss, cheek-to-cheek^a^	Cumprimento os meus amigos mais chegados com um beijo na face^a^
15	I feel comfortable touching people I do not know very well^a^	Sinto-me à vontade ao tocar em pessoas que não conheço muito bem^a^
16	I feel disgusted when I see public displays of intimate affection	Sinto-me enojado(a) quando vejo demonstrações íntimas de afeto em público
17	It would make me feel anxious if someone I had just met touched me on the wrist	Sentir-me-ia ansioso(a) se alguém que tivesse acabado de conhecer me tocasse no punho
18	If I had the means, I would get weekly professional massages^a^	Se tivesse condições, todas as semanas fazia massagens com um profissional^a^
19	I hate being tickled	Detesto que me façam cócegas
20	I like petting animals^a^	Gosto de fazer festas a animais^a^

^a^Items scored in reverse

### Phase 2—Reliability and Validity Test of the Portuguese Version of the STQ

#### Participants

For reliability and validity assessment, a total sample of 242 Portuguese university students was selected (59 % were students of physiotherapy and 41 % of speech therapy and occupational therapy) from volunteers to participate in the study. The choice of college students as the sample type followed the example of the original study.

The majority of the sample is female (83.1 %) and the mean age of the entire sample is 21.3 ± 3.8 (min = 17; max = 45) years. The sample size was in accordance with recommendations in the literature on the number of participants required for a factor analysis: more specifically, between four to ten subjects per questionnaire item with a minimum number of 100 subjects to ensure stability of the variance–covariance matrix (Kline 1993). The questionnaires were distributed to students in class and they were asked to register the total amount of time taken to complete the questionnaire. All participants returned the questionnaire. Test–retest reliability was performed with a smaller student sample (*n* = 50) over a two-week interval (Terwee et al. 2007). None of the participants reported any psychiatric or psychological condition or anxiolytic medication. Table 3 shows the sample characteristics.

**Table 3:** Sample characteristics (*N* = 242) and completion time of STQ

Age (years)^a^	21.31 ± 3.8 (max = 45; min = 21)
Female: male (*n*; %)	201 (83.1 %): 41 (16.9 %)
Physiotherapy (*n*; %)	143 (59 %)
Speech therapy and occupational therapy (*n*; %)	99 (41 %)
Completion time (min)^a^	2.92 ± .71 (max = 2; min = 5)

^a^Mean ± SD (max; min)

#### Reliability

The internal consistency was assessed using Cronbach’s α coefficient. Test–retest reliability was performed with a smaller student sample (*n* = 50) and assessed using Intraclass Correlation Coefficient (ICC). An ICC higher or equal to .70 is considered positive as long as the sample is composed of at least 50 subjects.

#### Validity

The construct validity is determined by how the score of an instrument relates with other measurements. This relationship must show consistency with theoretically derived hypotheses concerning the concepts involved in the study. In light of the relationship between social anxiety and avoidance behaviors towards touch described in the literature, we selected the European Portuguese version of the Social Interaction and Performance Anxiety and Avoidance Scale (SIPAAS) as a comparison measure (Pinto-Gouveia et al. 2003). Permission was given to use this scale.

It comprises two subscales, namely the distress/anxiety subscale and the avoidance subscale, and it is a self-report questionnaire to assess the level of distress and avoidance in a large variety of social performance and interaction situations. Both scales showed high levels of internal consistency. Total scores may range from 44 to 176 and the authors suggest cut-off scores (distress/anxiety subscale—115; avoidance subscale—105), thus discriminating between subject with generalized social phobia and the non-clinical population.

The construct validity was assessed using the predefined hypotheses test (Streiner and Norman 2003; Terwee et al. 2007): (1) A positive correlation is expected between the total scores of the STQ and the anxiety and avoidance subscales of the Social Interaction and Performance Anxiety and Avoidance Scale (SIPAAS); (2) Physiotherapy students have fewer avoidance behaviors and attitudes towards social touch, when compared with speech therapy and occupational Therapy students.

The Pearson Correlation Coefficient and the *t* test for equality of two population means were used for the statistical analysis of the construct validity. A value of *p* ≤ .05 was considered statistically significant.

Both exploratory (EFA) and confirmatory factor analysis (CFA) were applied to test the unidimensionality of the questionnaire. EFA is first chosen because it does not set any constraints on the estimation of dimensions. In contrast, the number of latent factors in CFA must be previously determined and the items loading on each specific factor must be specified. The SPSS and AMOS version 22 were used for all statistical analyses.

## Results

The mean STQ completion time was 2.92 min, ranging from 2 to 5 min. All items were completed. To assess the floor and ceiling effects of the STQ, we analyzed the distribution of each item; no such effects were found (Table 4).

**Table 4:** Floor and ceiling effects

	*n*	Floor effect %	Ceiling effect %
STQ	242	.00	.00

As we can see in Table 5, the STQ showed adequate internal consistency (Cronbach’s α = .734) and the test–retest correlation with the STQ items revealed a high concordance between the tests over a two-week interval for a sample size of 50 students (ICC = .990; Lower Bound = .981; Upper Bound = .995).

**Table 5:** Reliability**—**STQ

	Cronbach α (*n* = 242)	ICC (*n* = 50)	Lower bound	Upper bound
STQ	.734	.990	.981	.995

The results showed a significant conceptual convergence between the STQ and the SIPAAS-Anxiety (*r* = .64; *p* < .0001) and SIPAAS-Avoidance (*r* = .59; *p* < .0001), with a positive correlation between measurements. However, it appears that the avoidance behaviors and attitudes towards social touch (measured with STQ) are more associated with the distress felt in situations involving performance and social interaction (measured with the SIPAAS-Anxiety subscale) than with avoidance situations of performance and social interaction (measured with the SIPAAS-Avoidance subscale). As such, the first pre-defined hypothesis that there is a positive correlation between the total scores of the STQ and the anxiety and avoidance subscales of the SIPAAS was confirmed (Table 6).

**Table 6:** Validity—STQ versus SIPAAS

	SIPAAS Anxiety total score	SIPAAS Avoidance total score
STQ total score		
*r*	.639*	.590*
*p*	.000	.000
*n*	242	242

* Correlation is significant at the .01 level

Physiotherapy students exhibited fewer behaviors and attitudes towards social touch than Speech Therapy and Occupational Therapy students (*p* < .0001). In fact, Physiotherapy students have a lower score in STQ (29.18 ± 8.66) than the students from the other two degree courses (37.77 ± 7.85). Thus, the predefined hypothesis was confirmed (Table 7).

**Table 7:** Validity—STQ versus course

Course	*n*	Mean	SD	*p*
STQ _ total score				
Physiotherapy	143	29.18	8.66	
Other	99	37.77	7.85	.000

Factor analysis using the principal axis extraction method with Promax rotation was performed on the 20-item scale to identify the underlying dimensions of the Portuguese version of the STQ (Matsunaga 2010). Promax rotation allows the factors to be correlated. The KMO measure of sampling adequacy showed a value of .785, higher than the suggested minimum of .6 (Tabachnik and Fidell 2013). Bartlett’s test of sphericity revealed a Chi square of 1001.4 (*p* < .0001) which rejected the hypothesis of the population correlation matrix being an identity matrix, thus validating the suitability of the EFA.

The number of factors was not restricted. To assure convergent validity, .4 was used as a loading cut-off. Items had to display a .2 loading difference with all other factors to ensure distinctive validity. Using these criteria, a solution of 3 or 4 dimensions was detected with 41 % and 47 %, respectively, of explained variance Chi square goodness of fit test shows that the reproduced correlation matrix is not significantly different from the observed matrix for the two solutions (*p* > .15). All the factors successfully attained eigenvalues higher than one, as recommended by (Pallant 2007).

The results of the EFA indicate that STQ can be conceptualized as either a 3 or 4-factor model, indicating that a one-dimension STQ is clearly unacceptable for the Portuguese population. A more accurate analysis of the last model shows that no item loads higher than .5 in Factor 4, and only one item loads between .4 and .5 (“STQ-16. *I feel disgusted when I see public displays of intimate affection*”), shedding doubt on this dimension which should therefore be discarded. The second highest loading in this dimension is for item “STQ-8. *I’d feel uncomfortable if a professor touched me on the shoulder in public*”, which loads higher in Factor 1, but does not ensure distinctive validity. Similarly, item “STQ-1. *I generally like it when people express their affection towards me in a physical way*” fails to have distinctive validity.

Results for the 3 factor solution are presented in Table 8. Some items are not significant because they load with values lower than .4 in all dimensions: “STQ-19. *I hate being tickled*”, “STQ-16. *I feel disgusted when I see public displays of intimate affection*” and “STQ-18. *If I had the means, I would get weekly professional massages*”.

**Table 8:** EFA**—**structure matrix

	Loadings	% Variance
*Factor 1: Dislike of physical touch*		18.8
3. I get nervous when an acquaintance keeps holding my hand after a handshake	.629	
7. It annoys me when someone touches me unexpectedly	.570	
2. I feel uncomfortable when someone I don’t know very well hugs me	.562	
10. I feel uncomfortable if I make physical contact with a stranger on the bus or subway	.561	
17. It would make me feel anxious if someone I had just met touched me on the wrist	.514	
8. I’d feel uncomfortable if a professor touched me on the shoulder in public	.504	
5. I feel embarrassed if I have to touch someone in order to get their attention	.481	
13. I would rather avoid shaking hands with strangers	.407	
19. I hate being tickled	.389	
16. I feel disgusted when I see public displays of intimate affection	.341	
*Factor 2: Liking of familiar physical touch*		13.9
11. I like being caressed in intimate situations	.757	
12. As a child, I was often cuddled by family members (e.g. parents, siblings)	.629	
14. I greet my close friends with a kiss, cheek-to-cheek	.602	
20. I like petting animals	.508	
9. I’d be happy to give a neck/shoulder massage to a friend if they are feeling stressed	.474	
18. If I had the means, I would get weekly professional massages	.347	
*Factor 3: Liking of public physical touch*		8.5
6. I consider myself to be a ‘touchy-feely’ person	.725	
1. I generally like it when people express their affection towards me in a physical way	.611	
4. I generally seek physical contact with others	.609	
15. I feel comfortable touching people I do not know very well	.458	

CFA was then performed to test the measurement model. CFA hypothesizes an explicit a priori model of the construct structure, estimates its parameters and examines whether this model is an adequate fit with the original data. The match between the hypothesized CFA model and the observed data is evaluated with different fit statistics [Chi square goodness of fit statistic (χ^2^), Normed Chi square (χ^2^/degrees of freedom), Root Mean-Square Error of Approximation (RMSEA), Comparative Fit Index (CFI) and Expected Cross-Validation Index (ECVI)]. An overall good model fit is verified by a normed Chi square lower than 2 and a RMSEA not higher than .05; when different models are being compared, the one with highest CFI and lowest ECVI is the best (Hair et al. 2010).

**Fig. 1:**
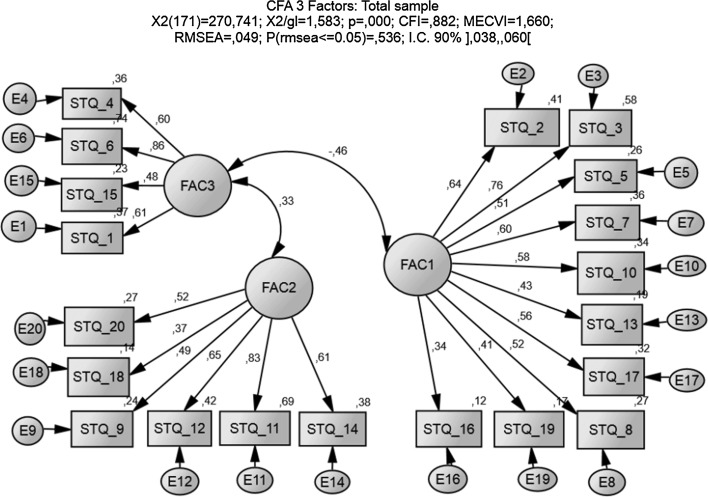
CFA—3 factor solution

CFA results for the null model (1-factor model) and comparisons with the 2 and 3 factors solutions are presented in Table 9. They show that the 3-factor solution generates the best fit indices and also that this model achieves a good fit of the observed data; it is presented diagrammatically in Fig. 1. The null model fit statistics indicate an unacceptable solution, refuting the uni-dimensionality of the STQ questionnaire. The overall fit of the 3-factor measurement model is good and shows a significant improvement over its lower factor counterparts.

**Table 9:** CFA fit indices—STQ 1, 2 and 3 factor solutions

Indices	1 Factor	2 Factors	3 Factors
χ^2^	613.73	380.48	270.74
χ^2^/*df*	3.589	2.212	1.583
CFI	.475	.753	.882
ECVI	3.036	2.060	1.513
RMSEA	.104	.071	.049

Confirming construct validity entails assessing convergent, discriminant and face validity. The presence of convergent validity can be assessed in different ways: significance tests for factor loadings and squared multiple correlation coefficients for each of the observed variables, average variance extracted (AVE), α Cronbach coefficient and composite reliability (CR) for each construct. Factor loadings should be significant and at least .50, preferably higher than .70; an AVE value higher than .50 indicates the construct is able to explain more than half of the variance of its observed variables; both α Cronbach and CR measure the internal consistency of a construct and must not be lower than .6. Discriminant validity is confirmed for each construct, with positive differences between AVE and the squared correlation of that construct with the other constructs, providing evidence of the uniqueness of each construct (Fornell and Larcker 1981). Face validity of the constructs is theoretically supported by the literature.

Results show that convergent validity is not assured. Although loadings are all significant (*p* < .001), the following observed items should be removed from the model due to their low standardized loadings: STQ-13, STQ-19 and STQ-16 in Factor 1; STQ-18 and STQ-9 in Factor 2; STQ-15 in Factor 3. AVE values are all lower than .5 (Table 10), showing that no factor is able to explain at least half of the variance of its observed variables; however, both Cronbach α and CR show acceptable levels of internal consistency for all factors. Here, AVE is bigger than the squared multiple correlation in all cases and gives evidence of the uniqueness of each construct.

**Table 10:** Validity testing—STQ 3 factor solution

Construct	Cronbach α	CR	AVE
Factor 1	.682	.804	.301
Factor 2	.712	.758	.356
Factor 3	.759	.738	.425

## Discussion

Our main goal was to evaluate the reliability and validity of the European Portuguese version of the STQ. The European Portuguese version of the STQ is easily understood and takes little time to complete. No floor and ceiling effects were found, revealing an excellent content validity.

We found a high level of reliability in the STQ; in fact, Cronbach’s α coefficient (.734) show that the internal consistency was acceptable, indicative of a high correlation among the items in the questionnaire and that the items are suitable to evaluate behaviors and attitudes towards touch. However, this value is slightly lower than the one reported by the original authors (.89). This result may be due to the fact that the original study sample consists of subjects with higher levels of anxiety. The Cronbach’s alpha coefficient is an inherent property of the studied population response pattern, not a feature of the scale alone; i.e., the alpha value undergoes changes according to the population to which the scale is applied (Streiner 2003). The STQ demonstrated excellent reproducibility, showing homogeneity in concept measurement and stability between evaluations over time.

The specific hypotheses established for construct validity were corroborated:

1. There is a positive correlation between the total scores of the STQ and the anxiety and avoidance subscales of the SIPAAS. The total score of the STQ is significant and positively correlated with the total scores of the anxiety and avoidance subscales of the SIPAAS, which supports the use of the STQ as a screening tool. This correlation was also found in the original study (Wilhelm et al. 2001) and there are other studies that corroborate this association (Nuszbaum et al. 2014). It means that social anxiety is related to a generalized pattern of anxiety and avoidance linked to situations involving touch. In this sample, the SIPAAS-Anxiety subscale is more associated with the avoidance behaviors and attitudes towards social touch (STQ) than with SIPAAS-Avoidance subscale, for which the total score indicates the level of avoidance in performance and social interaction situations. This relationship is probably associated with the fact that the sample consists of healthy individuals and, as such, they may feel high levels of anxiety in certain situations but, as they do not avoid these anxiogenic contexts, they are able to deal with situations and tasks that cause distress.
2. Physiotherapy students exhibit fewer avoidance behaviors and attitudes towards social touch, compared with Speech Therapy and Occupational Therapy students. The Physiotherapy course is based on two core learning strategies: theoretical lectures and hands-on practice, reproducing real-life situations or in clinical placement. From the first year of the course, students experience various learning situations which require touching each other and touching patients. Touch represents the highest proportion of nonverbal behavior in the physiotherapists’ interventions (Roberts and Bucksey 2007) and the profession depends on manual skills. But what distinguishes Physiotherapy from most other professions is the bodily interactions with the patients and long treatment sessions. Physiotherapists use touch through hands-on techniques but also to positively influence their relationship with patients (Roberts and Bucksey 2007). The literature refers to these touch categories as instrumental touch (a deliberate physical contact necessary to perform a treatment strategy) and expressive or affective touch, (a spontaneous physical contact, not essential for the completion of a task) (Everett et al. 1995).

In different social contexts, touch, the amount of touch quantity and how often it is applied increases compliance and promotes interpersonal relationships (Bohm and Hendricks 1997; Guéguen 2004; Guéguen and Vion 2009). However, it can also cause anxiety and avoidance reactions and when this occus in a therapeutic context, it may lead to the discontinuation of the therapy relationship. Therefore, it is advisable to evaluate the patient’s perception of touch through objective evaluation measures rather than on the basis of the therapist’s feelings (hunch). In this case, the STQ may be considered an important indicator to assess the therapeutic relationship.

Results from the exploratory analysis suggested that there are 3 multi-item factors for the Portuguese version of the STQ: Factor 1—Dislike of physical touch (10 items); Factor 2—Liking of familiar physical touch (6 items) and Factor 3—Liking of public physical touch (4 items). Furthermore, in line with the results for this specific population, some of the items should be removed: STQ-13, STQ-19 and STQ-16 from Factor 1, STQ-18 and STQ-9 from Factor 2 and STQ-15 from Factor 3. However, for future validation processes with other populations and contexts, a conceptual explanation is required before a decision is made to remove any original items from the questionnaire. Moreover, if the sample comprises adults not attending school, the item “STQ-8. *I’d feel uncomfortable if a professor touched me on the shoulder in public*” should be excluded. In other words, it may be necessary to adjust the original questionnaire to each specific population.

The main limitation in this study is that the sample is mostly female, and it was therefore not possible to determine the differences between men and women in relation to social touch. We recommend the replication of this study, using either a larger sample or clinical samples.

## Conclusion

The results of this study showed that the European Portuguese version of the STQ is a reliable, valid and comprehensive measurement tool. It is an instrument that can be used by different health professionals, in clinical practice and for research purposes, especially in studies that include touch experiences in their protocols whether they are tactile sensory stimuli applied passively or involving the haptic touch (when the subject actively explores and interacts with objects or other people).

## Footnotes

1 Individuals with mental disorders may experience significant distress in certain social situations and can even demonstrate social disability (American Psychiatric Association 2013).

2 Hands-on strategy is a common term used in Physiotherapy and it means an intervention where physiotherapists use their hands in direct contact with a patient’s body.
